# Translations from Foreign Exchanges

**Published:** 1883-10

**Authors:** 


### Translations from Foreign Exchanges.

TRANSLATED BY A. LAGORIO, M.D.

---

### ETIOLOGY OF CROUPOUS PNEUMONIA.

Drs. Griffin and Cambria, of Messina, have reported their experimental studies on croupous pneumonia. They have studied the blood in pneumonia, the liquids of cultivation of the bacillus of the blood, the sputum of pneumonia, and the action of the common irritants. From this series of observations and experiments they have drawn the following conclusions:

1st. That in the blood and in the sputum of pneumonia exists a special bacillus, different from Klebs' monads.

2nd. That the sputum of pneumonia, free of any saliva, when injected under the skin and in the trachea of rabbits and dogs, invariably produced in all a disease having the anatomical and clinical characters of acute septicæmia, always with a deadly result, and the blood of these animals, taken many hours (33) before death, when injected under the skin of another, rapidly killed him. The deadly action of the sputum was not modified by washing the mouth with an alcoholic solution of salicylic acid.

3rd. That while the saliva of pneumonia rapidly killed rabbits, in dogs it only produced an abscess in the place of puncture.

4th. That the defibrinated blood of pneumonia, whenever injected in the peritonæal cavity and in the trachea of rabbits and dogs, produced a variable elevation of temperature, less significant in the dogs, but never any form of pneumonia.

The fibrine of this blood, injected under the skin, killed a rabbit, but produced no action in a calf.

5th. That the liquids of various cultivations of the bacillus of the blood of pneumonia, injected under the skin and in the trachea, when this bacillus was in the various stages of vegetation, produced irregular elevations of temperature in rabbits and dogs, but no alterations of any sort in the lungs, nor in other organs. That the elevations of temperature were stronger in the rabbits when the liquid was of first cultivation, and the blood of these rabbits, containing the same bacillus, when injected in others, produced the same elevations of temperature. One drop of this liquid of cultivation, injected in the anterior chamber of the eye of a rabbit, produced a strong local suppurative inflammation, and death by septic infection.

6th. That the bacillus of the system and of the blood of pneumonia is transmitted always to inoculated rabbits and dogs, in whose blood it can be found, in a moderate quantity during life, and increases after the death of the animal. But this bacillus, transmitted in the blood of the animals, does not produce a croupous pneumonia, and its existence in the blood of pneumonia is to be considered a secondary fact; it is probably introduced in the lungs of pneumonia patients by the air, is rapidly and abundantly multiplied, and passes into the blood.

7th. That strong ammonia injected in small doses through the trachea in the lungs of dogs, and especially if previously infected with liquids of cultivation and with the blood of pneumonia, invariably produce a form of croupous pneumonia, and always lobar if the animal lived for a few days. A precedent infection with materials derived from pneumonia does not establish any predisposition in the animal; therefore the ammonia does not act in these cases as a cause simply occasional, but is capable, *per se*, of producing a true lobar croupous pneumonia. In our judgment, croupous pneumonia is not a disease of infection.—*Giornale Intern. delle Scienze Mediche.*

ALBERTONI—RESORCIN.

Resorcin, having a coagulating property for albuminoids, is recommended as a caustic for diseased tissues. When crystallized its cauterizing action is as strong as nitrate of silver, without pain, and without leaving colored cicatrices. Weak solutions are used to medicate wounds. It is employed in solution as an injection in diseases of the bladder. A solution of five per cent. of resorcin, used two or three times, is sufficient to cure any acute vesical catarrh, most often caused by gonorrhœa. In chronic conditions, the strength of solutions is 5 to 10 per cent.

Cutaneous affections are frequently cured by its use, as erysipelas, pemphigus, rupia, lepra. Resorcin is extremely useful in poisoned wounds by animals, or in lesions by instruments infected with cadaveric poisons.

Concentrated solutions can be employed to wash suppurative cavities and canals of the body, as the vagina; the œsophagus; the antrum of Highmore; the sinus frontalis.

Resorcin in a form of ointment (resorcin-glycerine-vaseline) acts with rapidity and surety in syphilitic infiltrations and ulcerations, in soft or hard sores, and in phagedenic ulcers of the genital apparatus.

It is used in gastro-enteritic affections, as cholera infantum, septic enteritis, dysentery, typhus. Audeers recommends resorcin in a solution 1-5 per cent., as a wash in chronic diseases of the stomach, because it is less poisonous than phenol and salicylic acid, and is hemostatic.

The antipyretic action of resorcin has been well studied by Lichteim and by Jänicke. According to Jänicke, doses of 0.50 every half hour, to 4 grams, lower the temperature from 1 to 1½ degrees C., which lasts two or three hours without causing any disturbance. The lowering of the temperature rarely lasts more than two hours; sometimes the temperature rises higher than the primitive limit.

To prepare resorcinated gauze, take 15 grams of resorcin, 450 grams of alcohol, 150 grams of glycerine, to every kilogram of gauze. The resorcinated lint is prepared by taking 30 grams of resorcin, 100 grams of alcohol, and 70 grams of glycerine, to a kilogram of lint. In cases of cholera infantum, it is given at a dose of 10-50 centgr. in an infusion of chamomile; as an antipyretic to adults, at a dose of 2-5 grams in a day.—*Lo Sperimentale.*

STATISTICS OF RESECTION OF THE STOMACH.

Ceccherelli has gathered in various journals the cases of resection of the pylorus executed up to to-day, and has reported the following, their authenticity being ascertained:

1st. Torelli—For wounds of the stomach, cure.—*Bulletino delle Seienze Mediche d' Bologna*, page 556, 1878.

2nd. Cavazzani—For a tumor in the walls of the stomach, cure.—*Gazzetta Medica Italiana, Provinzie Venete*, 1879.

3rd. Pèan—For carcinoma of the stomach, death.—*Gazette des Hôpitaux*, 1879.

4th. Rydzgier—For carcinoma of the stomach, death.—*Przeglad Lekarsk*, 1880.

5th. Billroth—For carcinoma of the stomach, cure; death after four months from gangrenous peritonitis. This was the first case, and was much talked about.

6th. Billroth—For carcinoma of the stomach, death.

7th. Billroth—For carcinoma of the stomach, death.

8th. Wôlfler—For carcinoma of the stomach, cure.

9th. Billroth—For carcinoma of the stomach, cure.—*Wiener Med. Woch.*

10th. A Surgeon of Arras—For a tumor of the stomach, death.—*Revue des Sciences Médicales*, vol. xvi, page 742, 1880.

11th.—Langenbeck—For carcinomatous stricture of the pylorus, death.—*Central-blatt für Chirurgie*, No. 20, 1881.

12th. Bardenheuer—For carcinoma of the stomach, death.—*Die Drainirung der Peritonealhöle*, 1881.

13th. Leuenstein—For a tumor of the stomach, death.—*Centralblatt für Chirurgie*, No. 29, 1882.

14th. Lücke—For carcinoma of the stomach, death.—*Deutsche Zeit. für Chirurgie*, 1882.

15th. Rydzgier—For a tumor of the stomach, cure.—*Centralblatt f. Chir.*, 1882.

16th. Hahn—For ectasis of the stomach, death.—*Centr. f. Chir.*, 1882.

17th. Czerny—For a tumor of the stomach, cure.—*Arch. f. Klin. Chir.*, 1882.

18th. Czerny—For carcinoma of the stomach, cure.

19th. Langenbeck—For carcinoma of the stomach, including also the stomach, death.—*Centr. f. Chir.*, 1882.

20th. Gussenbauer—For carcinoma of the stomach, death.

21st. Esmarch—For ulcer of the stomach, cure.—*Petersen Inang. Dissert.*, *Kiel*, 1882.

22nd. For carcinoma, death.—*British Med. Journal*, 1882.

23rd. Caselli—For partial stenosis of the orifice of the pylorus caused by carcinoma, death.— *L'Italia Medica*, 1882.

In these twenty-three cases collected by the author, nine cures are numbered; it is valuable to notice that death in the most cases occurred in a few hours after the operation by shock. In the last surgical congress held in Berlin, Billroth said that the greatest danger resided in the casual lesion of the pancreas. He also said that better results will be obtained in cases of dilatation of the stomach, and pyloric ulcers, than in carcinomatous stenosis of the pylorus.—*Lo Sperimentale.*

SULPHO-TARTRATE OF QUININE GLYCYRRHIZATED WITH COFFEE.

Dr. C. Pavesi says that in therapeutics every novelty provokes enthusiasm, and often the majority of physicians receive with benevolence any unusual remedy, provided it be *in mode*, and is eulogized by the journals. He does not love the empirical multiplicity of remedies, nor the poetry of some curative methods which fall before the criticism of experience. Frankly speaking, the sulphate of quinine, of all the alkaloids of the barks, is the oldest, and the most used in the medical practice of all nations. Considering that for its intense bitter taste it cannot be administered to many persons, and especially to children, he has masked the bitter taste with licorice root and toasted coffee, without in the least subtracting its precious therapeutical effects. The following is the method of preparation:

| | | |
|---|---|---|
| ℞. Sulphate of quinine, | parts | 1 |
| Tartaric acid, | " | 1 |
| Powd. of licorice root | " | 5 |
| Powd. toasted coffee, | " | 25 |
| Water, Q. S. | | |

The coffee and the licorice are mixed with sufficienthot water; the obtained liquid, limpid and brown, is evaporated to a syrupy consistency; then are added the sulphate of quinineand the tartaric acid, well mixed and dissolved; and again the whole is evaporated to dryness by slow heat so as not to alter the aroma of the coffee and the other immediate extractive principles. Being hygrometric, is kept in ground bottles. *Properties:* The sulpho-tartrate of quinine glycyrrhizated with coffee is a brownish powder resembling slightly coffee; it is of a bitter-sweet taste not disgusting, soluble in water. Tested with the proper re-agents the quinine is detected unaltered. It is to be administered in all the morbid affections, where quinine is indicated, especially to children. The syrup of sulpho-tartrate of quinine with licorice is prepared by dissolving in the decoction of coffee and licorice fifty parts of sugar and one part of sulphate of quinine and tartaric acid; then is evaporated by slow heat to the consistency of a thick syrup. It has a sweet taste, slightly bitter, very convenient for children. Every twenty-three grams of syrup contains about half a gram of sulphate of quinine, and as much tartaric acid.—*Gazzetta Medica di Torino; Il Morgagni.*

IODOFORM IN LUNG AFFECTIONS.—Prof. M. Semmola again calls attention in the *Giornale Internazionale delle Scienze Mediche* to the good effects of iodoform obtained in caseous broncho-alveolitis. He does not pretend to have discovered a specific for incipient tuberculosis, or for any caseous process of the lungs. He has been the first to advocate iodoform in these affections, and having obtained most excellent results, he warmly begs his colleagues to try it. According to his clinical experiences, the treatment with iodoform exercises in caseous broncho-alveolitis, or in incipient phthisis, the following effects: The expectoration diminishes rapidly and considerably. At the same time the cough lessens—becomes less distressing, probably on account of the local anæthesia produced by the remedy. The products which exist in the bronchi or in the cavities are disinfected. The fever progressively lessens, for the remedy disinfects and diminishes the putrid substances, which become absorbed by the softened parts. There is also a bettering of the local process, and in some cases is noted a beginning of a cure. Evidently the general state of the patient is improved, and in cases of caseous broncho-alveolitis in the first stages, a complete cure can be obtained.

Equally good results have been produced in bronchial catarrh, asthma, bronchiectasis, etc., all of which have been vouched for by Dr. Ciaramelli, Prof. Bufalini and Dr. Rumun, who have published them in the *Siècle Médical.* The daily dose is 5 centigr. to 40-50 cgm., and is to be regulated according to the tolerance of the digestive organs and the nervous system. The pillular form with the extract of gentian is to be preferred. Dr. Semmola gives small doses every hour. When the gastro-enteric apparatus is not in good condition, the iodoform is inhaled with the essential oil of turpentine three to four times a day.

### Cobweb in Intermittent Fever.

Oliva has published in the *Gazzette Médicale de Seville* statistics of twenty-six cases of intermittent fever of a quotidian and tertian type, cured with the tincture of cobweb. This medicament is prepared in the following manner: Having gathered a quantity of cobwebs, it is shaken sufficiently to separate the powder that it contains. This powder, of a grayish color, without smell, tasteless, is insoluble in water, and little soluble in alcohol. It is just the alcoholic solution which is used. The author has administered it frequently at a dose of two grams to adults, and of a gram to children. A boy seven years old took thirty grains of it in a few days, without experiencing the least phenomenon that could be attributed to the remedy. According to Dr. D. Delgrado y Ramos, the dose of three grains has produced symptoms of gastric intolerance and a marked cephalalgia.

Generally, two doses are sufficient to arrest the paroxysms. One hundred and nineteen observations have been published, and from these the following conclusions can be drawn: That the cobwebis an agent capable of curing palustral fever of a quotidian or tertian type. It is of no benefit in the quartam form. That when administered in a dose of two grams to adults and of one gram to children, it usually stops the fever at the second paroxysm. That its action being less rapid than that cf sulphate of quinine, it should not be given in pernicious fevers. That its want of taste renders it of more easy administration than quinine, especially to children.—*Il Morgagni.*

---

RESORCIN IN DIPHTHERIA.—Dr. Wm. Lee said he had recently had ten cases of diphtheria in the vicinity of Perkin's Square, the oldest subject being six years, in which he had found the following prescription very efficacious, a small quantity being placed upon the back of the tongue every half hour: ℞. Resorcin., gr. x; acid. tannic., ʒi; acid. salicylic., ℥ss; acid. boracic., ʒij; sulph. flav,, ℥ss. M. It is remarkable how rapidly the membrane becomes softened by it. An emetic is also useful because swallowing of the membrane causes irritation of the stomach. He objected to mopping the throat, which he thought did more harm than good by abrading the tonsil. He also gives the resorcin internally, as: ℞. Resorcin, gr. xxiv; aquæ, ℥vi. M. S. Tablespoonful every four hours for an adult. It acts as a tonic, but he also gave whisky and food. The cases were very malignant. In one the throat trouble disappeared, ear-ache set in and the child died of otitis media.—*Maryland Med. Journal.*

AMERICAN ACADEMY OF MEDICINE.—The annual meeting of the Academy will be held at the New York Academy of Medicine, 12 W. 31st street, New York, on Tuesday, October 9, (three o'clock P. M.), and Wednesday, October 10, 1883.

RICHARD J. DUNGLISON,
*Secretary.*

THE present number contains an account of the fatal illness of the promising young Dr. Frank L. Rea, and also contains his maiden contribution to medical literature.

